# The China tuberculosis clinical trials consortium network: a model for international TB clinical trials capacity building

**DOI:** 10.1186/s40249-020-00671-w

**Published:** 2020-05-15

**Authors:** Yu-Hong Liu, Shu Chen, Jing-Tao Gao, Yao Zhang, Kimberly Booher, Xiao-Fen Ding, Wei Shu, Jian Du, Jing Bao, Richard Hafner, Carol D. Hamilton, Liang Li

**Affiliations:** 1 Beijing Chest Hospital, Capital Medical University/Beijing Tuberculosis and Thoracic Tumor Institute, No. 9, Beiguan Ave, Tongzhou District, Beijing, 101149 China; 2 FHI Clinical, 359 Blackwell Street, Suite 200, Durham, 27701 USA; 3 Brii Biosciences, 3rd Floor, Building #7, Zhongguancun Dongsheng International Science Park, No. 1 North Yongtaizhuang Road Haidian District, Beijing, China; 4 U.S. National Institutes of Health (NIH), 5601 Fishers Lane Room 9E30, MSC, Bethesda, 9830 USA; 5 Duke University Medical Center, 359 Blackwell Street, Suite 200, Durham, NC 27701 USA

**Keywords:** Clinical trial consortium, Tuberculosis, Capacity building, China

## Abstract

**Background:**

With the second largest tuberculosis (TB) burden globally, China is committed to actively engage in international TB clinical trials to contribute to global TB research. However, lack of research capacity among local sites has been identified as a barrier.

**Main text:**

The China Tuberculosis Clinical Trials Consortium (CTCTC) was initiated by Beijing Chest Hospital with investment from the US National Institutes of Health and technical support from Family Health International 360 in 2013, as a nationwide collaborative clinical trial network to strengthen selected clinical site research capacity and attract TB clinical trials. The program aims to: 1) recruit leading hospitals that care for TB patients; 2) conduct on-site assessment to identify capacity gaps and needs for improvement; 3) design and deliver capacity building activities; 4) attract and deliver high quality results for TB clinical trials.

A total of 24 sites have joined CTCTC, covering 20 provinces in China. Twenty-two sites have been accredited by the National Medical Products Administration (NMPA) to be qualified to conduct TB clinical trials. The onsite assessment, extensive trainings among the CTCTC sites and young investigators have resulted in better understanding and improvement of the site capacity in conducting TB clinical trials. The establishment and growth of the CTCTC network has benefited from the good leadership, effective international cooperation and local commitment. Issues in human resources, regulatory environment and sustainability have been challenging the network from continuing growth. Clinical researchers have full-time clinical responsibilities in China and it is thus important to build a cadre of other human resources to assist. The regulatory environment is becoming friendlier in China to introduce international clinical trials to the CTCTC network.

**Conclusions:**

The CTCTC, with mature management structure and sustainable development model, which are distilled five key lessons for other developing countries or investigators of interest. They are the respectively using assessment-based approach to design tailored training package, understanding the availability of clinical researchers, providing solutions to maintain sustainability, understanding local regulatory environments and working with an international organization with local on-site team, respectively. Although, the experiences and capacity of China’s TB hospitals in conducting clinical research vary. Considerable efforts to continue building the capacity are still needed, although the gap is smaller for a few top-tier hospitals.

## Background

Tuberculosis (TB) is one of the top 10 causes of death worldwide and globally an estimated 10 million people developed TB disease in 2018, among which about 0.5 million people developed multidrug/rifampicin-resistant (MDR/RR) TB [1]. The development of new diagnostics, vaccines and drugs becomes paramount to control the epidemics, especially that of MDR/RR-TB. Building new tools requires multiple high-quality clinical trial sites with experienced investigators and good patient access. China alone accounted for 9% of global new TB cases and 14% of MDR/RR-TB cases in 2018 [1]. With good access to TB patients, China is committed to actively engage in international TB clinical trials to contribute to the global TB research development. However, with limited experiences in conducting international TB clinical trials, there has been high demand for capacity building in China as well as other TB high-burden developing countries. Through collaboration between Beijing Chest Hospital (BCH), the US National Institute of Health (NIH) and Family Health International (FHI) 360, the China Tuberculosis Clinical Trial Consortium (CTCTC) was established in 2013 to build a TB clinical trial network and strengthen the capacity among sites in the network. The specific objectives of this article are to: 1) summarize the establishment, achievements, challenges, lessons and future development of the CTCTC program in China; 2) disseminate the unique experiences and lessons learnt to other TB high-burden developing countries which may have plan to establish similar network and capacity building program; 3) bring CTCTC to the international TB community for future collaborations.

### Establishment of the China tuberculosis clinical trial consortium

Having identified that capacity building is key to preparing the sites to conduct TB clinical trials, Beijing Chest Hospital, which also serves as the Clinical Center of TB (CCTB), Chinese Center for Diseases Control (China CDC), initiated the CTCTC program in 2013. It was established as a nationwide collaborative clinical trial network in China with a primary mission to strengthen sites’ capacity and attract external funding for TB clinical trials. In addition to internal funding from BCH and other CTCTC member hospitals, the initiative was also invested in by the National Institute of Allergy and Infectious Diseases (NIAID), the US National Institute of Health through FHI 360, which provided technical support and guidance for clinical trial capacity building. The establishment of CTCTC gained enthusiastic and dedicated cooperation from local Chinese TB hospitals and investigators.

### CTCTC network and management structure

**Fig. 1:**
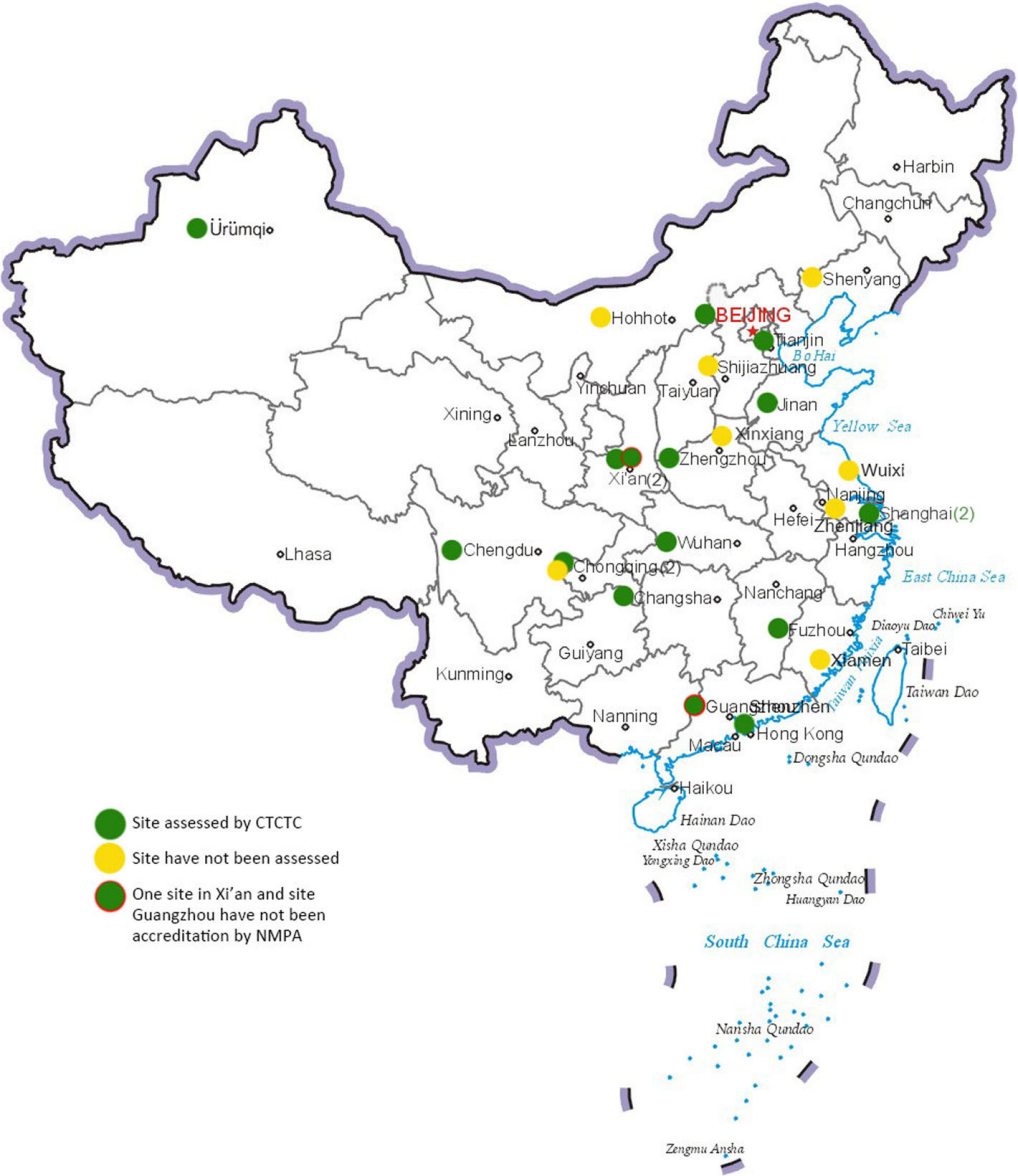
The CTCTC sites map as of March, 2019

The CTCTC started with 12 TB hospitals serving as the first group of potential clinical trial sites, selected in part because they had already been certified by former China Food and Drug Administration (CFDA, re-institutionalized to National Medical Products Administration, NMPA in 2018) to conduct TB clinical trials. This decision was key because, though many TB hospitals/clinical centers expressed interest to join the network, hospitals are not allowed to perform clinical trials without FDA certification for specific therapeutic areas, like TB [2]. CTCTC has doubled its number of sites during the past 5 years and the 24 sites cover 20 out of 34 provinces in China; only two of the 24 sites have not been accredited by NMPA (Fig. 1; Additional file 1 for the full list). It currently serves as the largest nationwide collaborative clinical trial network that brings together investigators, ethicists, community and other partners to test the safety and efficacy of drugs, vaccines, and diagnostics designed to prevent and cure TB. Several large scale domestic clinical studies are being conducted in CTCTC network, and it is poised to begin contributing international multicentre studies soon.

There are three levels of management for the CTCTC, including the Director Board, the Coordination Center and five Technical Units (Additional file 1). The Director Board is led by BCH/CCTB and consists of leadership from each CTCTC member hospital. The Board communicates through its annual meeting and ad hoc webinars to make decisions on critical issues related to CTCTC development. The CTCTC Coordination Center is based in BCH/CCTB, and coordinates CTCTC technical units, member hospitals and partners with responsibility for implementation of CTCTC’s annual workplans and activities. NIAID continues to play an important external consultant role, offering suggestions and advice when needed. There are five Technical Units which support the CTCTC. The Research Ethics Unit provides training, counselling and guidance for investigators to support ethical study design, ethical reviews and approvals for clinical trial/study/research. The Training Unit is responsible for implementing capacity building activities. The Scientific Panel comprises investigators who provide technical guidance in protocol design, implementation, quality management and data analysis. The Lab Unit provides technical support for TB laboratory related work and laboratory quality control (QC). The QC/monitoring and evaluation (M&E) unit supervises and evaluates the quality of clinical trials carried out by CTCTC.

### Capacity building design and implementation

With the goal to prepare the CTCTC sites for inclusion in registration-quality international TB clinical trials, BCH/CCTB worked together with the NIAID, NIH and FHI 360 teams to design an all-inclusive capacity building package. Three key principles were considered when designing the package, which were that it must a) contain the best international experiences; b) target the weaknesses of the sites; c) be sustainable. Based on these principles, the working team reviewed existing public-domain resources, including existing and previously US CDC and NIH-funded networks and research, research literature and documents. The team also consulted clinical trial research and implementation experts to summarize the best practices and lessons learned. An assessment-based approach was taken to identify the weaknesses and gaps of the sites to customize the capacity building plan. To increase the likelihood of sustainability, the package included a program targeting young investigators for research training and provided seed-funding to train young TB doctors in China to apply for and conduct high-quality TB medical research. Efforts are also ongoing to introduce international multi-center clinical trials to CTCTC sites that have demonstrated readiness, so that they can gain hands-on experiences in such a setting.

Hospital site assessments have been conducted four times covering 16 hospitals between the network’s inauguration (October 2013) and April 2019. The results have informed design and refinement of the training package. The core capacity building package covers eight domains including scientific knowledge, International Council for Harmonisation-Good Clinical Practice (ICH-GCP), research ethics, laboratory, data management, quality management, pharmacy management and young investigators research training and funding. CTCTC developed a set of minimum standards for conducting clinical trials, a clinical quality management plan and a set of consortium-wide CTCTC Standard Operating Procedures (SOPs) describing training and future clinical site expectations. In addition, CTCTC actively worked with selected projects among the network to provide project-based need-driven capacity trainings. The package was delivered through workshops, on-site and online trainings (Table 1).

**Table 1:** List of China Tuberculosis Clinical Trials Consortium (CTCTC) delivered training, as of June 2019

Purpose	CTCTC delivered training	Training methods
Scientific knowledge	Ongoing or recent studies of new strategies, shorter regimens for drug sensitive tuberculosis (TB)	Online
	Common toxicities and their management in the treatment of multidrug resistant and extensive drug resistant TB	Online
	TB Clinical Trial Development	Online
	Positron Emission Tomography/ Computed Tomography in TB treatment	Online
	Latest data sharing on treatment of drug-resistant TB	Online
	Expert Consensus on Essential Components of TB Treatment Trials: 2018 WHO Technical Consultation	Online
	Epidemiology study design	Workshop
	Clinical trial study design	Workshop
	TB clinical research: from theory to practice	Workshop
International Council for Harmonisation-Good Clinical Practice (ICH-GCP)	ICH-GCP and Informed Consent Workshop	Workshop
	Principles of ICH-GCP	Online
	ICH-GCP on-site training for 13 CTCTC sites	On-site
Research ethics	Research Ethics and Institutional Review Board Operations Training	Workshop
	Principles of Research Ethics	Online
	Step by Step for IRB Registration and instructions for Filing a Federal-wide Assurance	Online
Laboratory	Good Clinical Laboratory Practice (GCLP) Workshop	Workshop
	Global Microbiology Standard for TB clinical trials	On-site training
	Microbiology of clinical trials	On-site training
	CTCTC lab expert panel training	Workshop
	CTCTC lab core team GCLP training	Workshop
	University of North Carolina Laboratory & North Carolina State Laboratory Training	On-site training
	Molecular diagnostic progress of Drug resistance TB	Online
	Introduction to GCLP	Online
	STREAM (The Evaluation of a Standard Treatment Regimen of Anti-Tuberculosis Drugs for Patients with MDR-TB) Trial Lab Training	Project-based workshop
Data management	Data management in clinical trial/research	Online & workshop
	Principles of Biostatistics and Data Management in Clinical Research Workshop	Workshop
	Case Report Form design and database set up	Online
	Data quality management and monitoring	Online
	Safety data management and monitoring	Online
	Clinical data collection	Online
Quality management	CTCTC Minimum Standards for Conducting Clinical Trials	Online
	Didactic Monitoring Training	Workshop
	Risk-Based Approach to Monitoring	Online
	Inspection findings in Clinical trial	Online
	Quality control (QC) in clinical trials	Online
	MOST (Ministry of Science and Technology) study (A Randomized Multicenter Clinical Study of Ultra-Short Course Chemotherapy for New Smear-Positive Pulmonary Tuberculosis) Quality assurance (QA) training- Protocol Violation in clinical trial	Project-based workshop
	MOST study QA&QC team monitoring training	Project-based workshop and online training
	MOST study on-site QA mentoring training for QA staffs	Project-based onsite training
Pharmacy management	Pharmacy and study product management	Online
Young investigators research training and funding	CTCTC youth clinical investigators scientific workshop (trained 14 TB doctors so far) and small grants for the CTCTC investigators (15grants awarded) to conduct research projects	Workshop

### Facilitators and barriers

Establishment of the CTCTC and implementation of the capacity building activities could not have been successful without strong leadership, key partnerships and good commitment of the member sites. The CCTB, the lead institution of CTCTC, is located in BCH and also functions as the lead technical unit supporting China CDC and National Health Commission which in turn provides guidance for national TB diagnosis, treatment and clinical management within the national TB control programs. This access to national health policymakers and TB hospital resources has made BCH/CCTB an optimal choice to lead such an initiative. BCH also serves as the chair institution of the China Medical Association TB Society, and as such has used the CTCTC experience to establish a Clinical Trial Expert Committee within the TB Society to guide uptake and implementation of results from recent TB clinical trials and provide capacity building trainings.

Another key to the CTCTC’s success has been the effective collaboration between our international partners, including the NIAID, NIH and FHI 360, and the CTCTC leaderships and member hospitals. NIAID, NIH has kindly co-funded the program through FHI 360 and high-level strategic and technical support when needed. FHI 360’s on-site team provided daily technical support and assistance as well as effective communications with the BCH/CCTB leadership team, paramount to ensure smooth implementation of the program. Thanks to such international cooperation, the CTCTC network have access to international TB research laboratory expertise, and to senior advisors with extensive experience in clinical trials design and implementation. In addition, local CTCTC sites were strongly committed to improving their capacity to conduct clinical trials and actively participated in the organized trainings.

An ongoing barrier to success for the CTCTC is lack of a stable cadre of trained TB hospital personnel with time and interest to engage in TB research. Although there are staff at the hospital expected and committed to doing research, they generally have full-time clinical responsibilities and their time for research becomes quite limited. In addition, many young physicians and nurses switch to other specialties at the first opportunity because of better pay and lower risk of exposure and disease acquisition. The high turnover led to numerous cases where the physicians received capacity building training but soon left the TB department, which inevitably diminishes the returns on investment.

Regulatory hurdles are a big challenge when trying to introduce international TB clinical trials in China. For example, despite tremendous effort by CTCTC leadership, NIH/NIAID project leaders and FHI 360 senior scientists, the STREAM II trial [https://clinicaltrials.gov/ct2/show/NCT02409290] did not make it to China at the very final step in 2018 due to regulatory hurdles encountered during filing for a co-sponsored clinical trial with NMPA. The two major hurdles were that the STREAM II study required import of WHO-approved TB regimen background drugs (which were not China NMPA-tested and approved) and China’s restrictions on export of clinical TB isolates. Importing foreign drugs that have Chinese manufacturers, such as Moxifloxacin, are only allowed with the NMPA-issued clinical trial approval. In the STREAM II case, Janssen Pharmaceutical Company obtained the drug listing approval letter for bedaquiline, which cannot serve to introduce other background regimen TB drugs to China. In addition, international multi-center clinical trials typically require export of key biological samples so that they can be tested in a central laboratory to avoid any potential misdiagnoses and ensure consistent analytical results across study sites. Unfortunately, for decades now this has also been strictly forbidden under the current Chinese regulatory environment. The export of any human bio-sample out of China is scrutinized by the Human Genetic Resources Office, Ministry of Science and Technology and requires complicated and onerous administrative procedures. Therefore, coming up with an alternative solution for conducting such analyses locally is important if such trials are to include Chinese study sites.

The program also faces the sustainability issue. Although the CTCTC was funded by multiple parties including the BCH, other CTCTC member hospitals and NIAID, NIH, the ending of NIH’s funding poses challenges to the future development of the network. Nevertheless, the CTCTC leadership has come up with a few effective solutions. First, all the CTCTC hospitals have been invited to join the Innovation Alliance of TB Diagnosis and Treatment (Beijing) (IATB), a non-profit organization established by BCH to improve the capacities of TB hospitals in TB diagnosis and treatment. CTCTC has continued to grow with the flatform and resources provided by IATB. For example, IATB have worked with the industry to provide grants to CTCTC member hospitals and bring domestic clinical trial opportunities to the network. In addition, BCH has worked closely with FHI Clinical (former Department of Global Research Services, FHI 360) to plan international capacity building activities and seek for external funding opportunities.

### Collaboration opportunities and the way forward

Although there have been regulatory hurdles, the good news is the NMPA has started to take actions to relax the regulatory environment to fuel and expedite research and development in China. As of July 27, 2018, the NMPA has implemented a 60-working-day review period for clinical trial applications to expedite the review and approval [3]. If the NMPA does not raise any objections within 60 working days of the application fee payment, the study is permitted to move forward per the submitted protocol. It also allows data that has been generated from non-China clinical drug trials to be used for the application of registration, as long as they are consistent with China drug registration requirements, to facilitate “global new drug” applications [4]. Further, the registration application of new drugs to treat life-threatening diseases and disease of public health significance, such as TB, can be prioritized and go through the special “green channel” review [5]. In addition, since January 1, 2018, regulations guiding research of new medical devices have changed such that hospitals do not have to be accredited by NMPA to be qualified to do research for each type of device research, but are authorized to conduct clinical trials if they have met certain standard requirements recording in the system of NMPA [6]. It is expected that such regulation change will soon made to new drugs clinical trials as well [7].

#### Recent Progress and developments

With nearly 5 years of robust training, many of the CTCTC sites have been equipped with necessary theorical knowledge to serve as a TB clinical trial site. The first bedaquiline efficacy and safety implementation study aiming to recruit 1000 MDR-TB patients covering all provinces of China is being conducted in the CTCTC network. Several multinational and local manufacturers have approached CTCTC to test their new TB drugs, including bedaquiline, delamanid and pretomanid and other locally innovative drugs, from Phase I to IV clinical trials. The CTCTC leadership are also actively working with sites to design TB clinical trials that can answer China-specific questions with high-quality evidence. From 2017, the CTCTC initiated the research grant to encourage young investigators in member hospitals to design and implement small-scale clinical research. A total of 15 research projects from two rounds of (2017 and 2018) call-for-application were selected to be support with the CTCTC grant. With the regulatory environment becoming friendlier, it is also good timing for the international TB community to test the TB new drugs, vaccines and diagnostics on the CTCTC platform.

## Conclusions

In conclusion, the CTCTC, with mature management structure and sustainable development model, will continue to grow by absorbing new sites and building capacity to nurture world-class TB clinical trial sites and conduct high-quality clinical trials to contribute to the TB prevention and control. We summarized five key lessons learnt based on the five-year experiences of running the CTCTC program, for other developing countries or investigators interested in TB clinical trial capacity building. First, It is important to take the assessment-based approach so that tailored training can be provided to the specific site to make the most use of the resources invested. It is also suggested start with a few sites first, and expand the network when the program staff are more experienced. Second, it is necessary to understand the workload and availability of clinical researchers in the country before starting the capacity building program. Third, sustainability issue should be taken into consideration at the design stage of the program. It is always the case that for developing countries with limited experiences in conducting high-quality international TB clinical trials, considerable efforts to continue building the capacity are still needed after the ending of the training program. Capacity building is a long-term process and sustainable funding mechanism, especially by working with the country’s government, is strongly suggested. Fourth, understanding the local regulatory environment to introduce international clinical trials is essential. If there are foreseen barriers, it is suggested work with local authorities to find out solutions as early as possible. Fifth, working with an international organization with local on-site team is important to ensure successful and effective international cooperation. Having a local on-site team that understand both the western world and the local context, such as the FHI 360’s Beijing team, can help solve communications barriers brought by language and cultural difference. It is also worth note that if there are multiple parties or stakeholders, it is important to clarify each party’s role in this joint effort, especially when it comes to decision making.

Although, the experiences and capacity of China’s TB hospitals in conducting clinical research vary. The site capacity will be further strengthened through introducing clinical trials and organizing training activities, with support from local funding and possibly there international funders of interest to ensure its sustainability.

## Supplementary information

**Additional file 1.**

## Data Availability

Not applicable.

## Abbreviations

BCH: Beijing Chest Hospital
CCTB: Clinical Center on Tuberculosis, China CDC
CDC: Center for Disease Control
CFDA: China Food and Drug Administration
CTCTC: China Tuberculosis Clinical Trials Consortium
CRF: Case report form
FHI: Family Health International
FWA: Federal-wide Assurance
GCLP: Good Clinical Laboratory Practice
IATB: Innovation Alliance of TB Diagnosis and Treatment (Beijing)
ICH-GCP: Council for Harmonisation-Good Clinical Practice
IRB: Institutional review board
M&E: Monitoring and evaluation
MDR/RR-TB: Multidrug/rifampicin-resistant tuberculosis
MOST: Ministry of Science and technology
NIAID: National Institute of Allergy and Infectious Diseases
NIH: National Institutes of Health
NMPA: National Medical Products Administration
PET/CT: Positron Emission Tomography/Computed Tomography
QC: Quality control
SOPs: Standard operating procedures
STREAM: Standard treatment regimen of anti-tuberculosisdrugs for patients With MDR-TB
UNC: University of North Carolina
XDR-TB: Extensive drug resistant tuberculosis
