# Supplementary material for: The China tuberculosis clinical trials consortium network: a model for international TB clinical trials capacity building
Source: Infect Dis Poverty. 2020 May 15;9:52. doi: 10.1186/s40249-020-00671-w (PMC7229594; doi:10.1186/s40249-020-00671-w)

**Appendix 1**

**CTCTC member hospitals, as of June 2019**

|  | CTCTC member hospitals | City |
| --- | --- | --- |
| 1 | Beijing Chest Hospital | Beijing |
| 2 | Tianjin Haihe Hospital | Tianjin |
| 3 | Shanghai Pulmonary Hospital | Shanghai |
| 4 | Wuhan Institute for Tuberculosis Control | Wuhan |
| 5 | Changsha Central Hospital | Changsha |
| 6 | Chengdu Public Health Clinical Center | Chengdu |
| 7 | Shenzhen No.3 People’s Hospital | Shenzhen |
| 8 | Shandong Provincial Chest Hospital | Jinan |
| 9 | Fuzhou Pulmonary Hospital | Fuzhou |
| 10 | Henan Provincial Infectious Disease Hospital | Zhengzhou |
| 11 | Guangzhou Chest Hospital | Guangzhou |
| 12 | Shanghai Public Health Clinical Center | Shanghai |
| 13 | Shenyang Chest Hospital | Shenyang |
| 14 | Shaanxi Provincial Tuberculosis Institute | Xi’an |
| 15 | Zhenjiang No. 3 People’s Hospital | Zhenjiang |
| 16 | The 1st Affiliated Hospital of Xinxiang Medical College | Xinxiang |
| 17 | Wuxi No. 5 People’s Hospital | Wuxi |
| 18 | Xinjiang Chest Hospital | Urumuchi |
| 19 | The 1st Affiliated Hospital of Chongqing Medical University | Chongqing |
| 20 | The 1st Affiliated Hospital of Xiamen University | Xiamen |
| 21 | Hebei Provincial Chest Hospital | Shijiazhuang |
| 22 | Chongqing Public Health Medical Center | Chongqing |
| 23 | The No. 4 Hospital of Inner Mongolia | Hohhot |
| 24 | Xi’an Chest Hospital | Xi’an |

**Appendix 2**

**CTCTC Management Structure**


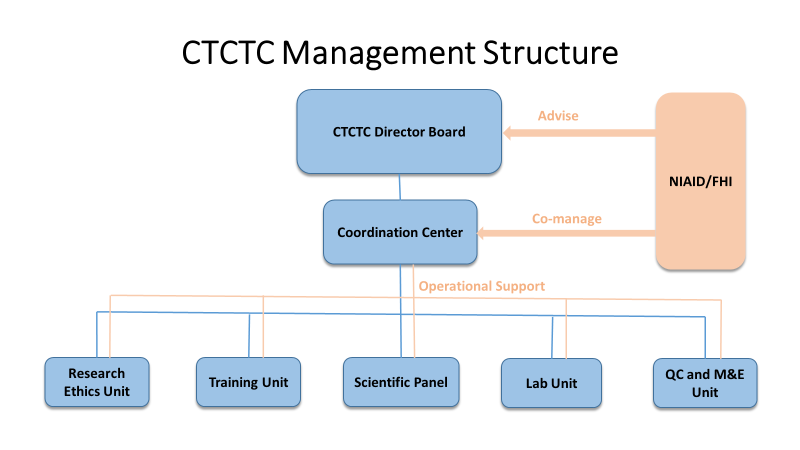

Supplement: Supplementary file 1 — Additional file 1. [file 40249_2020_671_MOESM1_ESM.docx]
